# A Post-donation Survey to Assess the Appropriateness of Medical Supply Donations to Freetown, Sierra Leone Following the Ebola Crisis

**DOI:** 10.7759/cureus.7228

**Published:** 2020-03-09

**Authors:** Alice Trye, Monica Maloney, Erica Jalal, Reshma Parikh, Samba Jalloh, Peter F Johnston, Vennila Padmanaban, Ziad Sifri

**Affiliations:** 1 Surgery, Rutgers New Jersey Medical School, Newark, USA; 2 Internal Medicine, College of Medicine and Allied Health Sciences University of Sierra Leone, Freetown, SLE

**Keywords:** global health, surgery, surgical supply donation, sierra leone, lmic

## Abstract

The Recovery of Equipment for Capacity building OVERseas (RECOVER) initiative at Rutgers New Jersey Medical School involves collection and donation of clean and unused medical supplies that would otherwise be discarded to those desperately in need of those supplies abroad. RECOVER has recently responded to the aftermath of the Ebola crisis and the even more recent mudslide natural disaster in Freetown, Sierra Leone, which had resulted in a considerable diminishing of the local medical supplies. The goal of this study was to assess the match between donated supplies and local needs by using a post-donation survey. In December 2016, we conducted a pre-donation survey inquiring which of the supplies available from RECOVER were needed by four hospitals in Freetown. The survey also asked about specific barriers to keeping such supplies in stock. After each hospital received a shipment of supplies, we administered an online Qualtrics (Qualtrics, Provo, UT) follow-up survey intending to assess the appropriateness of the donated supplies. The survey asked about which wards used what supplies, most useful items, ability to sterilize, and whether the donation provided supplies that would otherwise need to be bought. Recipient hospitals reported the use of 90% of donated supplies. The most useful supplies were gowns, scalpels, gloves, and drapes; All recipients reported the ability to sterilize donated goods. Supplies were used in operating rooms, emergency rooms, and medical wards. Donated supplies provided hospitals with supplies that would typically need to be bought or that were unavailable in the region. No adverse events were reported related to the use of donated supplies. At first glance, our donations appear usable and appropriate for the recipients. We hope to provide a framework for an objective measure of need for hospitals in other low-income countries, using the Freetown post-Ebola crisis as a pilot for the assessment of medical supply donations and the longitudinal impact it can have on global health and surgery overseas. More studies are required to further explore the possible implications of our program including those relating to medical waste management and environmental considerations when donating and shipping disposable supplies to a developing country.

## Introduction

Surgical supplies that are unused during a surgical procedure are destined for waste disposal every day in the United States. Due to strict hospital regulations and the overall medical practices of a litigious society, these items are deemed no longer fit for patient use. During any given surgical case, supplies may be prepared and kept ready for a procedure, yet often go unused by the end of the case. Although these supplies are still suitable for future use (i.e., their functional integrity is maintained and they can be re-sterilized), they are typically marked for disposal. However, it is well-known that these supplies are needed elsewhere. The Recovered Medical Equipment for the Developing World (REMEDY) project, implemented at Yale University School of Medicine in 1991, was one of the first attempts to organize a systematic collection and inventory protocol for unused medical supplies in the US intended for donation abroad [1-4]. In 2013, one group conducted a pilot study with two tertiary care centers in Guayaquil, Ecuador to assess the utility of donated supplies and assessed the disability-adjusted life years (DALY) averted as a result of donated supplies [5]. The Recovery of Equipment for Capacity building OVERseas (RECOVER) initiative at Rutgers New Jersey Medical School (NJMS) was originally started as a way to salvage clean and unused medical supplies destined for solid waste stream and donate them to low- and middle-income countries (LMICs) abroad. A few years later in 2016, RECOVER responded to the aftermath of the Ebola crisis in Freetown, Sierra Leone by donating a portion of the collected surgical supplies as the crisis was responsible for greatly diminishing the surgical capacity of regional hospitals [6,7]. 

Donation of medical and surgical equipment to LMICs overseas typically is a priority for a number of nonprofit and nongovernmental organizations (NGOs). However, regrettably, no system is in place to assess the appropriateness of donations to ensure they fit the needs of the recipient. Although the World Health Organization (WHO) has issued guidelines and detailed schematics for the donation of medications and equipment, donors rarely follow these guidelines [8]. The literature lacks significant evidence on the impact of survey tools in the donation of medical and surgical supplies to LMICs. 

Our goal was to assess the appropriateness of donated supplies with the needs of the participants by administering pre- and post-donation surveys to ensure appropriate donation of supplies. Our study looks at specific needs and barriers to surgical supply donations over a two-year period using pre- and post-donation surveys. We hypothesize that the survey and interview administration will lead to open communication between donors and recipients, decrease the number of unnecessary donations, and ultimately have a longitudinal impact on surgical supply donations overseas.

## Materials and methods

Study population

In December 2016, an in-person pre-donation survey was conducted at four hospitals in Freetown, Sierra Leone: Connaught Hospital, Princess Christian Maternity Hospital (PCMH), Police Hospital, and K’s Memorial Hospital. Connaught and PCMH were selected since they were the main hospitals in Freetown with accessibility to patients and established mechanisms of transporting donations. The other two were chosen based on the recommendation of stakeholders as they were newer hospitals with little access to surgical supplies from overseas. The survey focused on an initial needs assessment for available donations.

Survey schema

**Figure 1:**
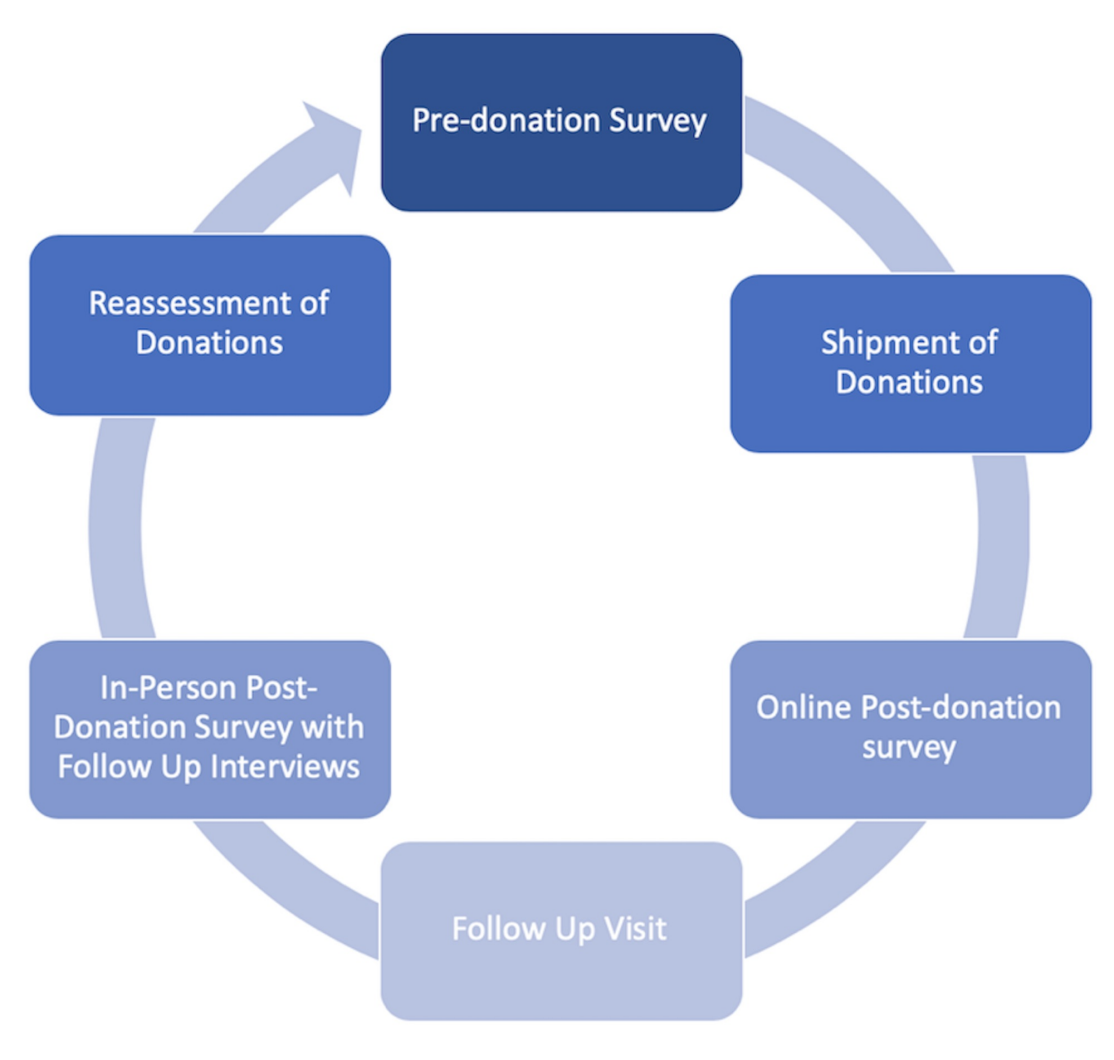
Survey schema

Three in-person interactions were conducted during the overall donation process: 1) pre-donation survey, 2) re-assessment and revisit while the shipment was in transit to maintain the connection between donor and recipient, and 3) post-donation survey on the received shipment, interview, and new pre-donation survey for the next shipment. A timeline of the survey and interview administration is depicted in Figure 1.

Pre-donation interactions

We conducted in-person interactions with stakeholders in December 2016 to assess the needs and barriers to surgical supplies (pre-donation survey questions are provided in Appendix A). In April 2017, a follow-up introductory meeting took place to aid continuity of communication with stakeholders and serve as a reminder for the in-transit shipment.

Packaging and shipping of supplies

We implemented WHO guidelines pertaining to surgical supplies collected from the operating room (OR), surgical ICU (SICU), and trauma bay of the donating hospital. Any surplus surgical supplies that were prepared and opened but ultimately unused for cases were deposited into RECOVER collection bins located in central areas of the donor hospital (within the operating room OR and SICU departments). Items were gathered and donated by nurses, residents, attendings, and other staff informed about the program and its purpose. Medical student volunteers from the affiliated medical school collected these items 1x week from both units. The students transported the donations to the school where they then weighed, sorted, and inspected all items. Students checked each item to ensure whether it matched hospital clinical care standards and whether it was suitable for clinical use abroad. Those items deemed suitable were subsequently inventoried and packaged for future shipments. Unfit items (expired, visibly soiled, broken, or torn) were weighed and discarded. Pre-donation meetings revealed all the recipients had the ability to sterilize donations and, therefore, items were not sterilized before being packaged. A continuous line of clear communication between the unit staff (responsible for item deposition) and the program volunteers (responsible for item collection, sorting, and evaluation) was essential in ensuring that the correct types of supplies in terms of both quality and category were being collected at the end of each case.

Survey tools

In June 2018, after the receipt of donations, an online validated Qualtrics follow-up survey consisting of 17-questions to assess the appropriateness of the donated supplies was administered. Our goal was to assess whether there was any equipment that was not being used, whether the donations increased the burden of waste in any way, and if each hospital had the resources and facilities to properly sterilize the supplies (i.e., autoclave and strip supply). The survey assessed the following parameters: the most useful items, supply utilization in the wards, the hospital’s ability to sterilize donated items, and whether the donations were useful.

Post-donation survey data collection and analysis

Data collection for post-donation was conducted in-person in June 2018. Stakeholders at Connaught Hospital and PCMH were also interviewed using standardized interview questions. Post-donation surveys and standardized interview questions are provided in Appendix B and C.

## Results

Demographics

The initial pre-donation survey recruited four hospitals to assess current supply needs. Table 1 provides detailed study population demographics. 

**Table 1:** Study population demographics

Hospital	Department	Type	Patient Population
Connaught Hospital	Surgery, Medicine	Urban Tertiary Center	800,000
Princess Christian Maternity Hospital	Surgery	Urban Tertiary Center, Urgent Care Center	2.5 million
K’s Memorial Hospital	Surgery	Community Hospital	20,000
Police Hospital	Surgery	District Hospital	50,000

Pre-donation survey results

Pre-donation survey results from recipient hospitals (n = 4) provided a focused list of supplies requested from each hospital (Appendix A). Out of the 18 supplies listed, an average of 16 were selected. All stakeholders reported the ability to sterilize donated goods but reported a shortage of autoclave indicator strips. Furthermore, the survey revealed that cost, unavailability of supplies, and demand exceeding available stock of supplies were primary barriers each facility faced.

Post-donation survey results

In post-donation surveys, recipient hospitals (n = 3) reported 90% use of supplies. The remaining 10% of unused supplies consisted of items not applicable to the operating theatre structure in Freetown (e.g., light handle covers) or not commonly used items at PCMH (e.g., bulb syringes). None of the supplies sent were considered damaged or unfit for use by recipients. Supplies not needed immediately were stored in the central medical stores of each institution. 

According to survey respondents, the most useful items were gowns, scalpels, gloves, drapes, surgical preps, 4x4 gauzes, gauze rolls, and laceration repair kits. Supplies were used in ORs, emergency rooms, and medical wards. Stakeholders confirmed that donated supplies provided both hospital locations with items that would otherwise need to be imported from other countries due to the lack of medical supply manufacturing within Sierra Leone. No adverse events were reported in relation to the use of donated supplies. 

## Discussion

Due to strict regulations in the United States, unused surgical supplies often go to waste despite being fit for use. With an appropriate needs assessment, these supplies can be donated to LMICs for use after sterilization. The WHO guidelines relating to this, published in March 2000, were proposed with the goals to maximize donations, minimize waste, and aid in the longitudinal assessment and impact of donations. Despite these guidelines, when international donors send medical supplies to LMICs, they do not routinely perform an assessment of need using pre- and post-donation surveys. The RECOVER initiative has adopted and demonstrated a successful example of the practical implementation of the WHO guidelines as a means of fostering a beneficial arrangement for the recipients of supplies in LMICs. 

Our surveys ensured that focused needs were met and revealed no adverse events related to donated supplies. Additionally, the surveys provided a mechanism of communication between donors and recipients, which facilitated conversations about potential unexpected concerns and assessed novel products that were needed yet unfulfilled by other sources. For example, because fabric surgical gowns are hung outside to dry, the operating theatres at Connaught can be closed for days to weeks during the rainy season. Through pre-surveys, our program was able to tailor our donations to include disposable gowns for this time period. 

Longitudinal surveys set a foundation for open and continuous communication to streamline the donation process. We provide a framework for an objective measure of need for hospitals in other low-income countries, using Freetown in its post-Ebola crisis state as a pilot for assessment of medical supply donations and the longitudinal impact it can have on global health and surgery overseas. The information can be used to identify key aspects of the donation pathway, including whether the recipients are able to retrieve donations once they reach the intended destination and identification of a reliable point of contact. Interviews revealed that there was no production of surgical supplies in Sierra Leone with the majority of medical equipment coming from outside the country. 

Survey responses revealed that donations provided items otherwise not available to the region. The items deemed “most useful” were all disposable, single-use items that have been documented in the literature to be the most wasted items here in the United States. Packaging and shipment of supplies were successful with no reports of damage in the shipping process. Shipment inventory and scheduling were adjusted based on survey results and interviews. Items that were not used, such as light handle covers and bulb syringes at PCMH, will no longer be sent since hospitals reported that the items were not useful; this type of information can be used to make future donations more successful as outlined in the WHO guidelines for post-donation follow-up. Through the post-donation survey, we now know that items that cannot be used at one institution can instead be shipped to other facilities that can make use of them. 

The longitudinal nature of these surveys allowed our program to provide a focused donation stream to fulfill the needs of the recipients. Additionally, structured interviews provided recipient hospital stakeholders with a platform to express concerns or appreciation in a format that allowed free communication. We believe this interview format demonstrated to them their role as an equal partner with a donation program that was considerate of their needs. Initial pre-donation surveys were completed with the four hospitals listed in Table 1. After the evaluation of the existing donation processing infrastructure, two hospitals (Connaught Hospital and PCMH) were chosen to complete post-donation surveys and follow-up interviews. Although four hospitals listed in Table 1 were originally administered the pre-donation surveys, three of them (PCMH, K’s Memorial, Police Hospital) received the first shipment of donations. Because K’s Memorial and Police Hospitals are newer hospitals with a lower number of patient populations, the decision was made to focus donation efforts on Connaught and PCMH based on the availability of supplies and larger patient volume. 

Limitations 

The major limitation of this survey was the small sample size. Another could be some of the survey responses that we suspect may have been influenced by the desire to please the surveyor. Additionally, we were unable to verify that the information provided was fully correct (i.e., issues pertaining to corruption and black market). 

Future directions

It is important to note that the majority of donation supplies were marked for single use. WHO recommends that donor nations/hospitals only donate items that match or exceed the standard of quality that is expected in the US. Single-use items destined for donation should be only used once and a written disclaimer regarding the same should be included with the inventory list and supplies. Interviews with stakeholders revealed that single-use supplies were often reused. According to WHO, donors are responsible for informing recipients of donations that the items being donated are labeled correctly and accurately. Hence, we recommend that a disclaimer relating to the same be included with all such future shipments.

## Conclusions

Pre- and post-surveys are powerful tools for reducing waste and are consistent with the WHO guidelines for healthcare equipment donations. Due to its clearly demonstrated success and lack of any apparent harm, pre-/post-surveys and stakeholder interviews should be conducted by all donors in accordance with the WHO recommendations. More studies need to be conducted to further explore the possible implications of our program, including, but not limited to, those pertaining to cost-effectiveness, waste/environmental considerations, and partnering with community organizations to streamline ground transportation of supplies to individual hospitals.

## Appendices

Appendix A: Pre-donation survey

1. Hospital/clinic name:

2. Location (city, country):

3. Contract name:

4. Type of facility (please check one):

District hospital

Urgent care center

Rural clinic

Urban tertiary center

Other: (please describe)

5. Estimated patient population served?

6. What is your major funding source?

Government-funded

Private organization

Charitable contribution

Other:

7. Which supplies are most in need? (Please check all that apply)

Drapes

Gowns

Suction tubing

Suction tips

Specimen containers

Laparotomy pads

OR towels

Basins

Surgical prep (e.g. betadine)

Loban

Light handles

X-ray detectable 4x4 gauze

Bulb syringes

Scalpel blades (sterile)

Surgical gloves (sterile)

Surgical gloves (non-sterile)

Bone wax suture (various types, sterile)

Back table covers

Mayo covers

Marking pens

Skin staplers

Stockinettes

Chest tubes

Endotracheal tubes (sterile, various sizes)

Oro/nasopharyngeal airways

Suction catheters (sterile)

Oxygen supplies (nasal cannula/face masks)

4x4 Gauze

Casting tape

Casting cotton/underpadding

Gauze roll

Coban

ACE bandage

Occlusive dressing (e.g., Tegaderm)

Laceration repair kit

Compression stockings

Lubricating jelly

Ultrasound gel

Needles (various sizes)

Angiocatheters

IV tubing

IV start kits

Tourniquets

Blood pressure cuffs

Disposable pulse oximeter sensors

Stethoscopes (disposable)

Arterial blood gas kits

Arterial line kits

Vacutainers phlebotomy needles

Saline ampules (10cc, sterile)

Other (please list)

8. Does your facility have a sterilizer/autoclave?          

Yes

No

9. What, if any, barriers does your facility face?

Cost

Availability

Use of materials outpaces supply

Shipment delays

Corruption/ theft of supplies

Other (please describe)

10. Does your organization/facility have no established transportation system for receiving donations from overseas?

Yes

No

Appendix B: Post-donation survey 

1. Did the donation provide you with the supplies that otherwise needed to be bought?

Yes

No

2. Did the donation provide you with supplies not available in your region?

Yes

No

3. Were the patients charged for any donated supplies?

Yes

No

4. Approximately what percentage of the donation was used? (Please check one)

<10%

10-25%

26-50%

51-75%

76-100%

5. Approximately what percentage of the donation could NOT be used? (Please check one)

<10%

10-25%

26-50%

51-75%

76-100%

6. How frequently would you prefer donations? (Please check one)

Every 3 months

Every 6 months

Once per year

As needed

Suggestion for improvement/wish list of items:

Appendix C: Interview questions 

1. How are supplies received and stored at the hospital? 

2. Are there any space limitations for donated supplies? 

3. How often does the government provide the hospital with supplies? 

4. If you need supplies where do you get it from? 

5. How often do patients have to pay for surgery? 

6. Does the government give money to cover those patients? 

7. How do you dispose of the donations and surgical supplies in general (mode of disposal)? 

8. How do you reprocess or sterilize the donated goods? 

9. How would you rank challenges/difficulties in obtaining equipment for the hospital? 

10.Most common surgeries?
